# Review of “Ascaris: *the neglected parasite*” by Celia Holland (Ed.)

**DOI:** 10.1186/1756-3305-7-33

**Published:** 2014-01-17

**Authors:** Rupert Quinnell

**Affiliations:** 1 School of Biology, University of Leeds, Leeds LS2 9JT, England

## Book details

Holland C, Ed: Ascaris: *the neglected parasite.* Academic Press; 2013. 439 pages. ISBN 978-0-12-396978-1

## Review

Ever since Norman Stoll’s estimate of the number of infections in 1947, *Ascaris* has been infamous as the most prevalent parasitic infection of humans, and plausibly the most prevalent of all human infections. Despite this, and notwithstanding the recent upturn of interest in neglected tropical diseases, *Ascaris* continues to be under-researched. Indeed, as suggested here, ascariasis has a good claim to be the ‘ultimate neglected tropical disease’. The publication of this book is thus very welcome, with its aim to highlight this state of affairs and to stimulate interest in this much neglected parasite.

The book is divided into five broad sections, covering Biology, Model Systems, Epidemiology, Host and Parasite Genetics and Clinical Aspects/Public Health. Within each section, the choice and focus of chapters reflects those areas with the most recent research activity, but overall the breadth and depth of coverage are excellent. Moreover, the efforts of a small number of dedicated researchers mean that, despite its neglected status, there has been significant recent progress in a number of areas. The Biology chapters provide first a detailed overview of what is known of immune responses in ascariasis – much of the research in this area being stimulated by interest in the potential effect of immunomodulation by helminths on allergic responses. This is followed by chapters on antigens and allergens, co-infection, and model systems for larval ascariasis. Together these chapters provide an excellent summary of the biology and immunology of the host-parasite relationship. The second Model Systems chapter has a different focus, reviewing the advances in nematode neurobiology that have been made using *Ascaris*, for which its sheer size makes it an invaluable model organism.

The next six chapters focus on epidemiology and genetics, from mathematical models to genome sequencing. These chapters include much recent, and some unpublished, material. In these areas, research on *Ascaris* is at the forefront of human nematode research, and topics covered include the use of modern statistical methods to explore variation in worm burdens, the relationship between human and pig parasites - whether they should be classified as separate species depending on one’s choice of species concept - and how the integration of host, parasite and spatial genetics can provide new insights into the epidemiology of infection. Genetic studies will be boosted by the recent sequencing of the *Ascaris* genome, where the size of the parasite also proved useful. These chapters also clearly explain how and why such studies should be carried out, including why helminthologists should be interested in parasite population genetics and phylogenetics, and how to sequence a nematode genome.

The final section takes us into the more applied aspects of clinical impact and public health. The two human-focused chapters provide an overview of the worldwide disease burden, with a valuable insight into the methods of the recent Global Burden of Disease study, and a progress report on disease control worldwide. There have been some control successes, such as the recent decline in *Ascaris* infections in China, but progress worldwide remains slow. Reading these chapters also highlights the lack of recent research on human pathology – there are some key unanswered questions posed in the foreword, and both pathological mechanisms and the resulting effects on health and development remain little studied. The other two chapters focus on the impact and control of ascariasis in pigs. Inclusion of research on *Ascaris suum* is a welcome feature of the book. Despite the close relationship between human and pig parasites, there has been relatively little interaction between human and veterinary studies. From a human perspective, these chapters highlight some obvious similarities, but also some intriguing potential differences, in the processes underlying epidemiology and disease burden in the two hosts.

In an era of rapid scientific advances and open access publishing, the need for books such as this is sometimes questioned. However, there are clear advantages in having such a breadth of information in a single volume. The editor and contributors have succeeded in producing an admirably up to date, detailed and well-referenced set of chapters, and the very neglect of *Ascaris* means that the book will remain topical for longer. There are very few errors, though a few figures are hard to read in greyscale (colour versions are available on the publisher’s website).

Overall, this volume can be highly recommended to all with an interest in parasitic nematode infection and neglected tropical diseases. It is both an excellent summary of the current state of knowledge of *Ascaris* infection, and a very useful resource for those studying other parasites. As the cover states, it ‘provides a blueprint of how a single parasite can stimulate interest in basic biology, clinical science, veterinary science, public health and epidemiology’, and will hopefully continue to stimulate interest for years to come.

## Competing interests

The author declares that he has no competing interests.

